# SGLT2 inhibitors preserve serum chloride in non-diabetic CKD: a propensity-matched and LASSO regression analysis

**DOI:** 10.1080/0886022X.2026.2624169

**Published:** 2026-02-09

**Authors:** Masaru Matsui, Takaaki Kosugi, Shunsuke Kitamura, Masatoshi Nishimoto, Akiko Itano, Marumi Yamamoto, Haruka Yabuta, Aiko Oda, Masato Kawakami, Hideo Tsushima, Keisuke Okamoto, Masahiro Eriguchi, Ken-ichi Samejima, Kazuhiko Tsuruya

**Affiliations:** a Department of Nephrology, Nara Medical University, Kashihara, Japan; b Department of Nephrology, Nara Prefectural General Medical Center, Nara, Japan

**Keywords:** Chloride homeostasis, renal tubular physiology, cardiorenal protection, electrolyte signaling, sodium–glucose cotransporter 2 inhibitor, non-diabetic chronic kidney disease

## Abstract

Sodium–glucose cotransporter 2 inhibitors (SGLT2is) are novel renoprotective agents for patients with chronic kidney disease (CKD) and have diverse effects, including on the regulation of electrolyte balance. However, their effects on serum chloride concentrations remain unclear. We conducted a retrospective single-center study of 343 CKD patients without diabetes or proteinuria who were not taking diuretics, including 202 SGLT2i users and 141 non-users, and applied propensity score (PS) matching and LASSO regression analysis. The outcomes were the change in chloride concentration with adjustment for covariates, before and after PS matching. Factors associated with these changes were identified using multivariable analysis and LASSO regression. An adjusted linear mixed effects model showed that the annual changes in chloride concentration for the non-SGLT2i and SGLT2i users were −0.39 (95% CI: −0.61 to 0.17) mEq/L/year and 0.49 (95% CI: −0.02 to 1.00) mEq/L/year, respectively [difference 0.88 (95% CI: 0.58 to 1.17) mEq/L/year] (*p* < 0.001). After PS matching, there was also a significant difference between users and non-users of SGLT2is in the mean change in chloride concentration [difference 0.44 (95% CI: 0.03 to 0.84) mEq/L/year] (*p* = 0.036). Subgroup analyses confirmed these findings. Furthermore, the use of SGLT2is had the strongest influence on the 2-year change in serum chloride concentration. To our knowledge, this is the first propensity-matched study to demonstrate a sustained chloride-preserving effect of SGLT2 inhibitors in non-diabetic CKD. In conclusion, this study identifies a previously underrecognized tubular electrolyte effect of SGLT2 inhibitors—preservation of serum chloride—which may partly explain their consistent cardioprotective effects across diverse CKD populations.

## Introduction

Chronic kidney disease (CKD) is a critical global public health concern, given that it is associated with higher risks of cardiovascular disease and progression to end-stage renal disease, as well as having substantial economic implications for healthcare systems worldwide [1,2]. Conventionally, the treatment of CKD has relied on renin–angiotensin system inhibitors, as well as lifestyle modifications with respect to nutrition and physical activity [3,4]. The emergence of SGLT2 inhibitors (SGLT2is) has markedly transformed the management of CKD, particularly because of their ability to delay disease progression and reduce the risk of incident heart failure [5,6]. However, in addition to their established cardiorenal protective effects, SGLT2is have been shown to confer a broad range of clinical benefits.

SGLT2is have been shown to reduce hepcidin concentrations, thereby enhancing iron utilization and increasing hemoglobin concentration, regardless of the presence or absence of anemia [7,8]. This effect significantly reduces the likelihood that new therapies for anemia will be required [9]. Serum uric acid concentrations are significantly reduced by SGLT2is owing to increases in the tubule luminal glucose concentration and the upregulation of GLUT9 [10,11], and this mechanism contributes to a lower risk of developing hyperuricemia or gout [12]. Furthermore, SGLT2is improve hepatic function by reducing hepatic fat accumulation and improve the lipid profiles of patients, including through reductions in triglyceride concentrations [13,14]. In experimental studies, SGLT2 inhibitors have been suggested to exert endothelial protective effects by preventing intracellular influx of sodium and glucose, as well as to confer mitochondrial protection, potentially through mechanisms mediated by ketone bodies [15,16]. SGLT2is are also known to have various effects on electrolytes, including a reduction in tissue sodium content and the prevention of hyperkalemia [17,18]. Recently, several studies have shown that SGLT2is increase serum magnesium concentrations [19,20]. In addition, SGLT2is increase tubular luminal sodium concentrations, thereby contributing to afferent arteriolar constriction through a stimulation of the macula densa [21], it has been hypothesized that, in the downstream segments of the tubule, compensatory enhancement of sodium reabsorption occurs in parallel with augmented chloride reabsorption. Thus, as shown by the two pilot studies [22,23], SGLT2is may increase serum chloride concentrations, but clear clinical evidence of this effect is lacking. Therefore, in the present study, we aimed to compare the changes in the serum chloride concentrations of patients with CKD but no diabetes or proteinuria who were SGLT2i users or non-users. The results identify a previously underrecognized tubular electrolyte effect of SGLT2 inhibitors, which may partly explain their consistent cardioprotective benefits across diverse CKD populations.

## Methods

### Patients

We recently performed a retrospective single-center study of 362 patients with CKD but no diabetes or proteinuria of >0.5 g/gCr [24]. The absence of proteinuria was determined based on a single measurement. In brief, 43 patients were excluded from an initial cohort of 405 patients with proteinuria <0.5 g/gCr: 11 who were unable to continue SGLT2i therapy because of adverse events, 11 who had missing baseline data, and 21 who were lost to follow-up. In the present study, an additional 18 patients who were taking diuretics at baseline or during the study period and one patient for whom multiple measurements of serum chloride concentration were not available were excluded, resulting in a final sample size of 202 SGLT2i users and 141 non-users. All the included patients received a multidisciplinary team-based educational intervention, as previously described [24]. The time of the multidisciplinary educational intervention was defined as the baseline. Patients who initiated SGLT2i therapy at this time were categorized as SGLT2i users, whereas those who did not receive SGLT2i were classified as non-users.

The study was performed in accordance with the principles of the Declaration of Helsinki and approved by the Ethics Committee of Nara General Medical Center (approval number 316). Informed consent was obtained using an opt-out approach, whereby information about the study was placed on the institution’s website and participants were given the opportunity to refuse participation (http://www.nara-hp.jp/about/ethics). The requirement for written informed consent was waived owing to the retrospective nature of the study.

### Outcomes

The outcome was the change in chloride concentration after adjustment for covariates. Subgroup analyses were performed to evaluate the changes in chloride concentration in groups stratified according to the median concentration, age, sex, and other relevant clinical variables. Comparable analyses were also conducted for the serum sodium and potassium concentrations. Furthermore, the changes identified were similar after propensity score (PS) matching was used to minimize the differences in the characteristics of the two groups.

### Statistical analysis

An on-treatment analysis was employed to assess the therapeutic efficacy of SGLT2is, with patients who discontinued treatment because of death or adverse events being excluded from the final sample. All the variables are expressed as median (interquartile range). Baseline characteristics were compared between the SGLT2 inhibitor users and non-users using standardized mean differences (SMDs) before and after propensity score matching. An SMD of <0.1 was considered to indicate adequate balance between groups. A mixed effects model with an unstructured variance–covariance structure was employed to compare the changes in chloride concentrations of the SGLT2i users and non-users, accounting for patient-level random intercept and slopes. We used an unstructured covariance matrix for the random effects to flexibly model the covariance between intercepts and slopes, allowing for individual variability in both baseline chloride levels and trajectories. The factors included in the model were age, sex, baseline eGFR, urine protein creatinine ratio (UPCR), body mass index, underlying kidney diseases, hypertension, current and previous smoking, baseline sodium concentration, baseline potassium concentration, baseline chloride concentration, and the use of renin–angiotensin receptor (RAS) inhibitors at visits 1 month before and after PS matching. PSs were calculated using multivariable logistic regression to estimate the probability of taking an SGLT2i. Demographics (age and sex), comorbidities and lifestyle (hypertension, dyslipidemia, body mass index, and smoking status), laboratory data (baseline eGFR, UPCR, and the sodium, potassium, and chloride concentrations), underlying kidney diseases, and the use of RAS inhibitors were included as covariates. Model fit was evaluated using information criteria and a likelihood ratio test. The final mixed-effects model showed acceptable fit (AIC = 5,326.8, BIC = 5,409.8), and the inclusion of random effects significantly improved the model compared with a fixed-effects model (*χ*^2^ = 176.3, *p* < 0.001). The covariates were also included in the PS model, in which PSs were used to match SGLT2i users to non-users at a 1:1 ratio using a greedy nearest-neighbor matching algorithm. To examine whether the effect of SGLT2 inhibitor use on the annual eGFR slope differed across subgroups, we tested the interaction between SGLT2 inhibitor use and each subgroup in multivariable linear regression models.

Multivariable logistic regression was used to identify factors associated with the change in chloride concentration. Furthermore, LASSO regression was performed to address multicollinearity among covariates, such as the eGFR 1 month before the commencement of SGLT2i treatment, the baseline eGFR, and the eGFR 3 months after commencing treatment, and to identify the factors that were most relevant to the change in chloride concentration. The optimal penalization parameter (*λ*) was selected according to the minimum value of the Bayesian information criterion. This approach permits variables to be selected while avoiding overfitting, thereby improving the generalizability of the model.

Two-sided *p*-values of <0.05 were considered to indicate statistical significance. STATA MP version 17 (Stata Corp., College Station, TX, USA) was used to perform the statistical analyses.

## Results

### Baseline characteristics of the patients

**Figure 1.**
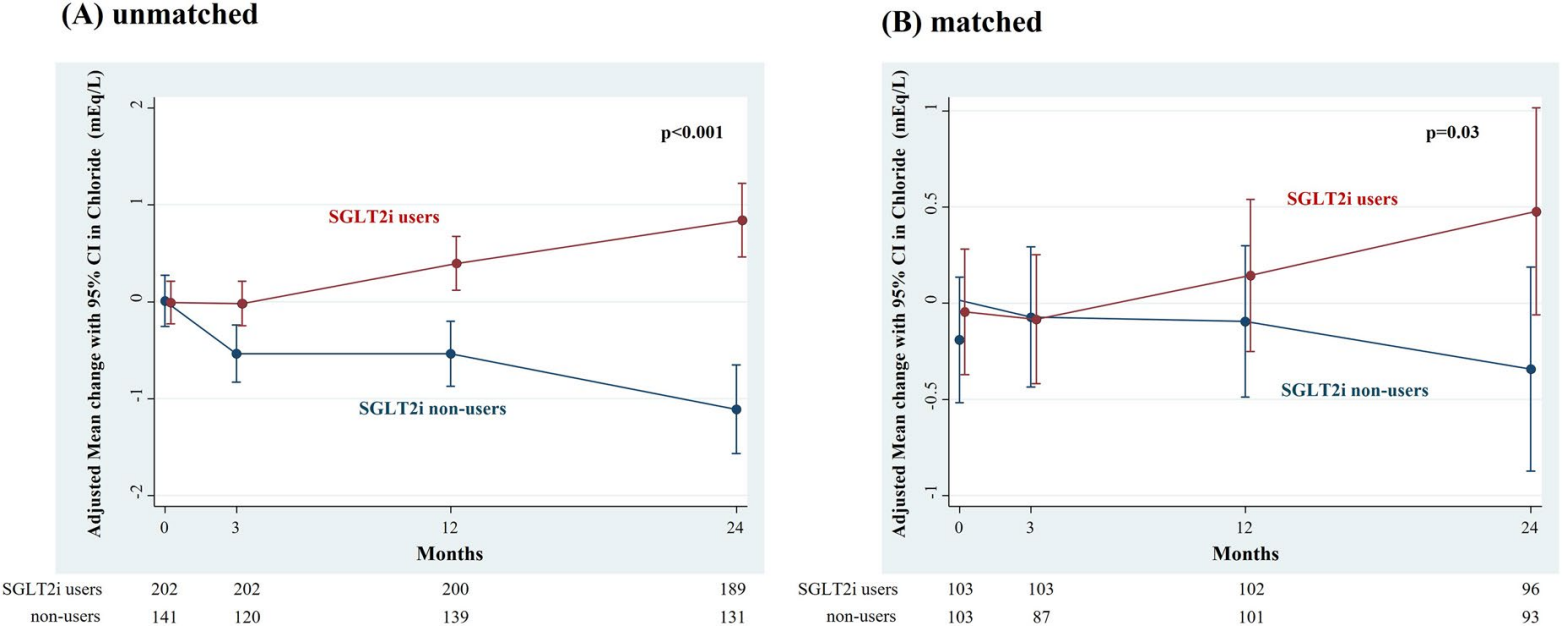
Adjusted change in chloride in SGLT2i users and non-users before (A) and after (B) PS matching. SGLT2i, sodium–glucose cotransporter 2 inhibitor; PS, propensity score. Over a 24-month period, the use of SGLT2 inhibitors significantly increased serum chloride concentrations compared to non-users, before (A) and after (B) PS matching.

We compared 202 SGLT2i users and 141 non-users who had CKD but no diabetes and UPCR <0.5 g/gCr (Figure 1). Their baseline characteristics before and after PS matching are listed in Table 1. Before PS matching, SGLT2i users were more likely to be male, had higher body mass index, had higher prevalences of chronic glomerulonephritis and hypertensive nephrosclerosis as underlying kidney diseases, and had lower serum chloride concentrations than non-users. However, after PS matching, the baseline characteristics of the two groups were very similar, as evidenced by SMDs and the Love plot (Supplementary Figure 1).

**Table 1.** Baseline characteristics of the patients before and after propensity score matching.

	Before PS matching		SMD	After PS matching		SMD
	SGLT2i users	Non-SGLT2i users		SGLT2i users	Non-SGLT2i users	
No. of participants	202	141		103	103	
Age, years	65 (54–72)	67 (51–75)	0.03	66 (55–72)	67 (51–75)	0.07
Sex (male), *n* (%)	128 (63)	72 (51)	0.25	63 (61)	56 (54)	0.12
Etiology of CKD						
Chronic glomerulonephritis, *n* (%)	75 (37)	37 (26)	0.47	22 (21)	32 (31)	0.10
Nephrosclerosis, *n* (%)	89 (44)	42 (30)		53 (51)	41 (40)	
Other, *n* (%)	38 (19)	62 (44)		28 (27)	30 (29)	
Body mass index, kg/m^2^	24.1 (21.8–26.3)	22.8(20.4–25.2)	0.35	23.8 (21.3–26.0)	23.4 (20.2–25.3)	0.11
Hypertension, *n* (%)	123 (61)	75 (53)	0.16	62 (60)	58 (56)	0.08
Dyslipidemia, *n* (%)	84 (42)	49 (35)	0.14	43 (42)	42 (41)	0.02
Smoker, *n* (%)	47 (23)	45 (32)	0.19	24 (23)	25 (24)	0.02
eGFR at baseline, mL/min/1.73 m^2^	45 (38–53)	45 (35–56)	0.16	45 (36–53)	43 (34–53)	0.07
eGFR 1 year before baseline, mL/min/1.73 m^2^	47 (39–53)	48 (37–58)	0.22	47 (39–53)	47 (36–56)	0.12
eGFR 3 months after baseline, mL/min/1.73 m^2^	43 (35–50)	45 (34–56)*	0.22	43 (35–51)	44 (35–55)	0.14
Proteinuria, g/gCr	0.15 (0.10–0.28)	0.16 (0.08–0.29)	0.08	0.14 (0.09–0.28)	0.17 (0.08–0.29)	0.01
Sodium, mEq/L	140 (139–142)	140 (139–142)	0.11	140 (139–142)	140 (139–142)	0.03
Potassium, mEq/L	4.2 (4.0–4.5)	4.2 (4.0–4.5)	0.15	4.2 (4.0–4.5)	4.2 (4.0–4.5)	0.06
Chloride, mEq/L	104 (102–105)	106 (104–107)	0.72	104 (103–106)	104 (103–106)	0.01
Renin–angiotensin receptor blocker, *n* (%)	113 (56)	65 (46)	0.2	52 (50)	50 (49)	0.04

PS, propensity score; SGLT2i, sodium–glucose cotransporter 2 inhibitor; CKD, chronic kidney disease; eGFR, estimated glomerular filtration rate.

* Data were missing for 13 patients.

#### Changes in chloride concentration

The median treatment duration was 2 years, during which clinical and laboratory parameters were monitored for all patients. Figure 1(A) shows the difference in the adjusted mean changes in chloride concentration between the SGLT2i users and non-users over a 2-year period. The unadjusted linear mixed effects model showed that patients taking an SGLT2i had a slightly larger annual change in chloride concentration [difference 0.94 (95% CI: 0.63 to 1.24 mEq/L/year)] than the non-SGLT2i users over the 2-year follow-up period (*p* < 0.001). In the SGLT2i users, the decline in chloride concentration was attenuated compared with the non-SGLT2i users, even in terms of absolute values. At baseline, the proportion of patients with chloride levels <104 mEq/L (the overall median) was 62% in the SGLT2i users and 37% in the non-SGLT2i users, which changed to 47 and 49%, respectively, after 24 months (Supplementary Figure 2). The adjusted linear mixed effects model showed that the annual change in chloride concentration for the non-SGLT2i users was −0.48 (95% CI: −0.71 to −0.25) mEq/L/year, compared to 0.45 (95% CI: −0.08 to 0.98) mEq/L/year for the SGLT2i users, corresponding to a difference of 0.93 (95% CI: 0.63 to 1.22) mEq/L/year (*p* < 0.001).

**Figure 2.**
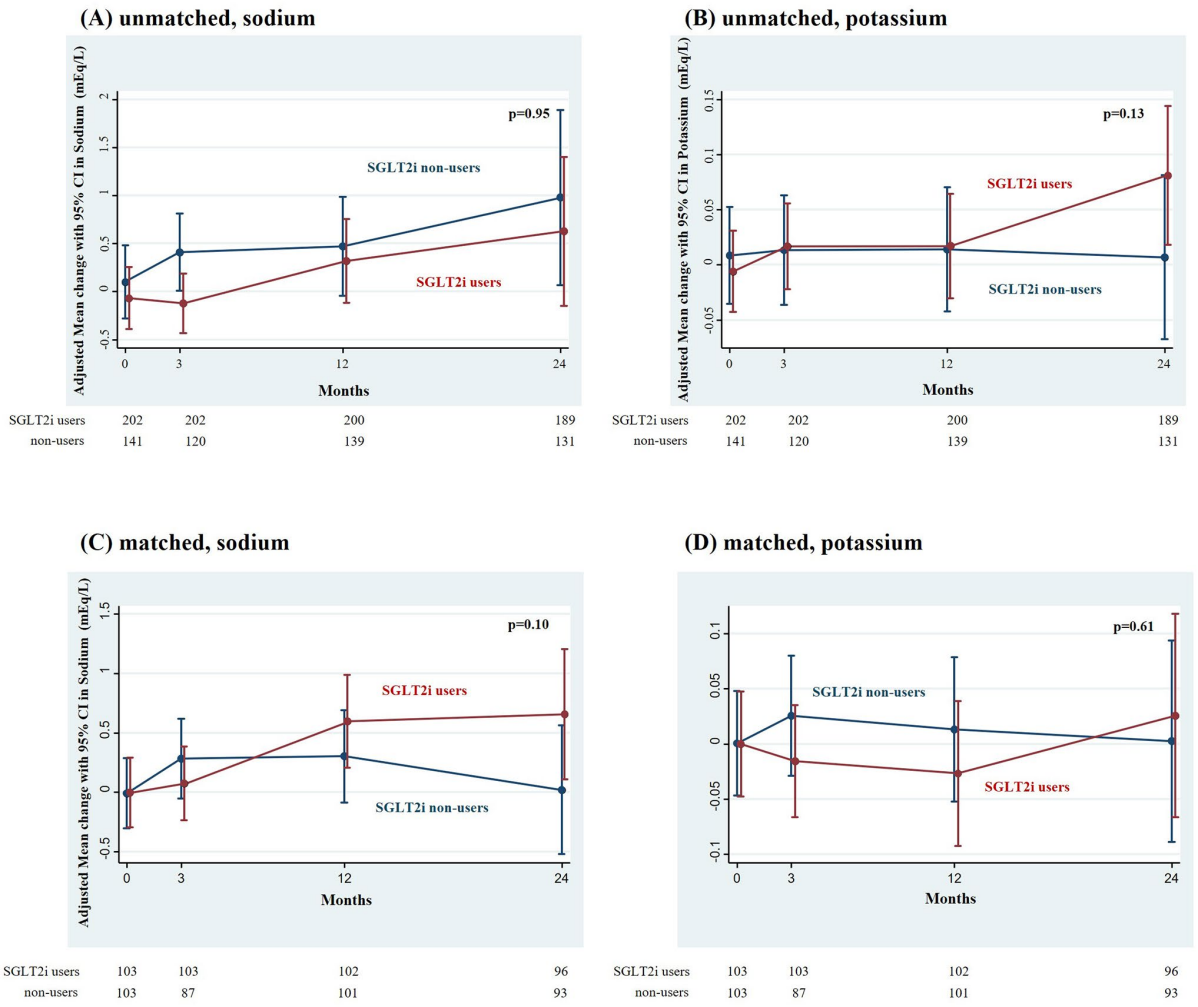
Adjusted changes in sodium and potassium concentration in SGLT2i users and non-users, before (A,B) and after (C,D) PS matching. SGLT2i, sodium–glucose cotransporter 2 inhibitor. There were no differences in mean changes in sodium (A,C) and potassium (B,D) between SGLT2i users and non-users, before (A,B) and after (C,D) propensity-score matching.

We then matched 103 non-users and 103 users of SGLT2is based on their PSs, and found a significant difference between the users and non-users of SGLT2is with respect to the mean change in chloride concentration [difference 0.44 (95% CI: 0.03 to 0.84) mEq/L/year)] (*p* = 0.03) (Figure 1(B)). Conversely, the differences in the annual changes in serum sodium and potassium concentrations between SGLT2i users and non-users were only −0.023 (95% CI: −0.73 to 0.69) mEq/L/year and 0.035 (95% CI: −0.01 to 0.08) mEq/L/year, respectively, and were not statistically significant (*p* = 0.95 and *p* = 0.13, respectively) (Figure 2). Similar results were observed in the matched cohort.

#### Subgroup analyses

**Figure 3.**
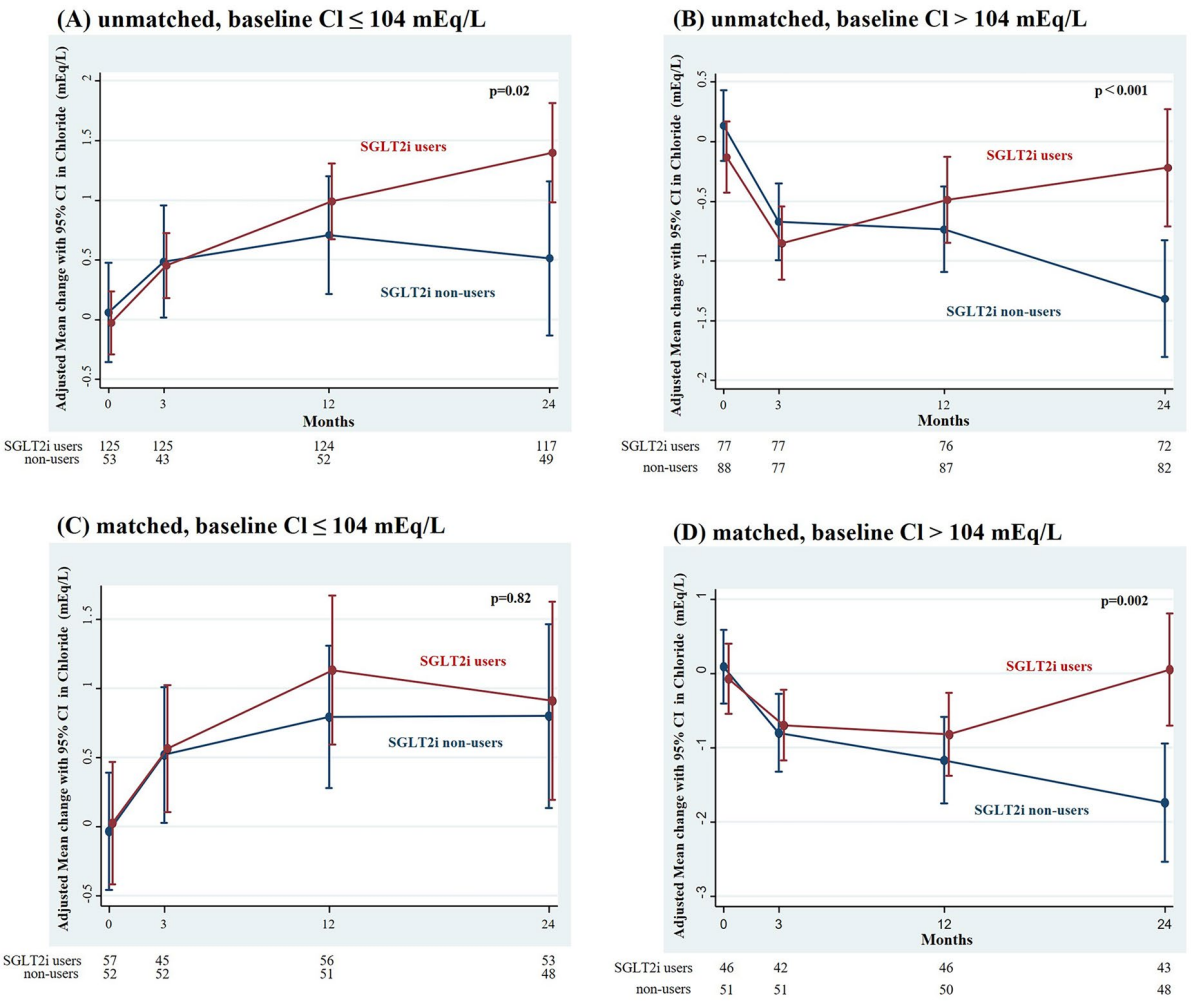
Adjusted change in chloride in SGLT2i users and non-users according to baseline chloride concentrations before (A,B) and after (C,D) PS matching. SGLT2 inhibitor users exhibited a significantly greater increase in serum chloride concentrations compared to non-users, both in patients with baseline chloride concentrations ≤104 mEq/L (A) and those with concentrations >104 mEq/L (B) before PS matching. After PS matching, there was no difference in chloride concentrations between SGLT2i users and non-users in patients with baseline chloride concentrations ≤104 mEq/L (C); however, in those with Cl >104 mEq/L (D), SGLT2i users maintained significantly higher chloride concentrations compared with non-users.

**Figure 4.**
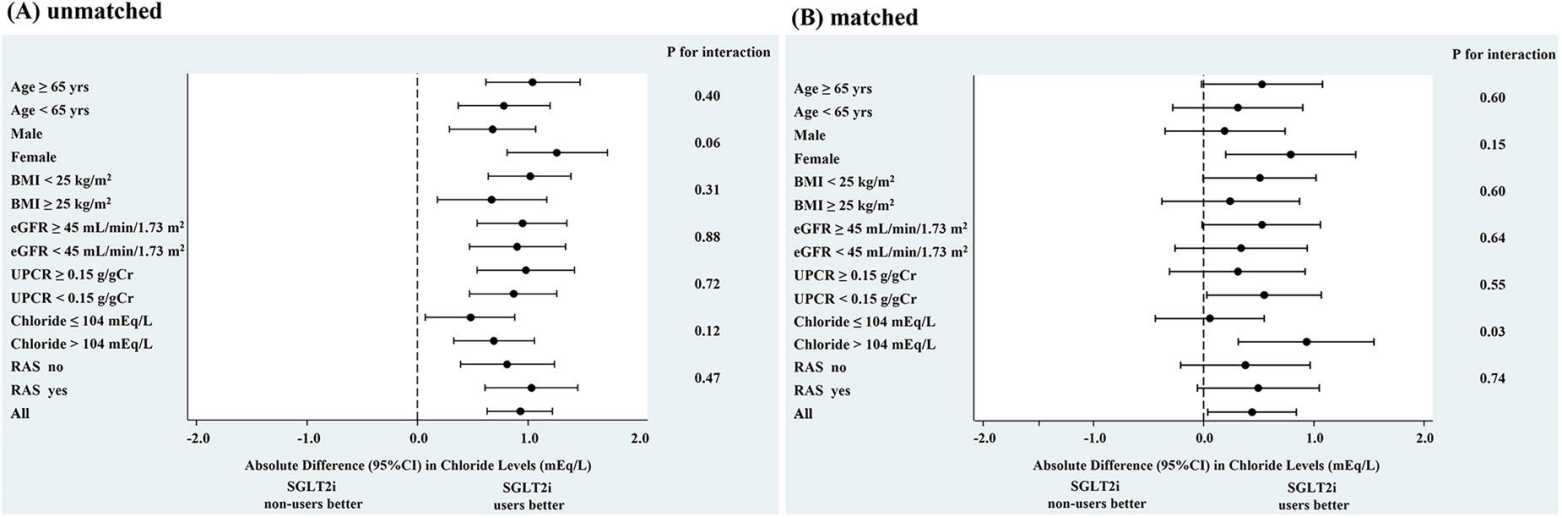
Adjusted change in chloride in SGLT2i users and non-users according to subgroups before (A) and after (B) PS matching. BMI, body mass index; eGFR, estimated glomerular filtration rate; UPCR, urinary protein-to-creatinine ratio; RASi, renin–angiotensin system inhibitors; CI, confidence interval; SGLT2i, sodium–glucose cotransporter 2 inhibitor. The mean changes in chloride concentrations were significantly larger in the SGLT2i users than in the non-users in subgroups stratified by age, sex, BMI, eGFR, UPCR, or RASi use before PS matching (A). After matching, these associations were attenuated, but the effects remained consistent across all subgroups except those stratified by baseline chloride concentration (B).

When the patients were categorized according to the median baseline chloride concentration of 104 mEq/L, we found that the group with lower baseline chloride concentrations achieved significantly larger SGLT2i-induced increases in chloride concentrations than non-users [between-group difference 0.48 (95% CI: 0.07 to 0.88) mEq/L/year; *p* = 0.02]. In contrast, in the group with higher baseline chloride concentrations (Figure 3), SGLT2i users showed a preservation of their chloride concentrations during the study period, resulting in a between-group difference of 0.69 (95% CI: 0.33 to 1.06) mEq/L/year (*p* < 0.001). In the matched cohort, however, no significant difference in the increase of chloride concentrations was observed between the two groups (*p* = 0.82) when baseline Cl was ≤104 mEq/L, whereas findings for Cl >104 mEq/L were consistent with those in the unmatched cohort (*p* = 0.002). The results of the other subgroup analyses of the changes in chloride concentration are shown in Figure 4. SGLT2i use was associated with a significant amelioration of the decline in chloride concentration *versus* those not taking an SGLT2i in the subgroups stratified by age, sex, body mass index, eGFR, UPCR, or use of renin–angiotensin system inhibitors. In contrast, although these associations were attenuated after propensity-score matching, the effects remained consistent across all subgroups, with the exception of those stratified by baseline chloride concentration.

### Factors associated with the changes in chloride concentration

**Figure 5.**
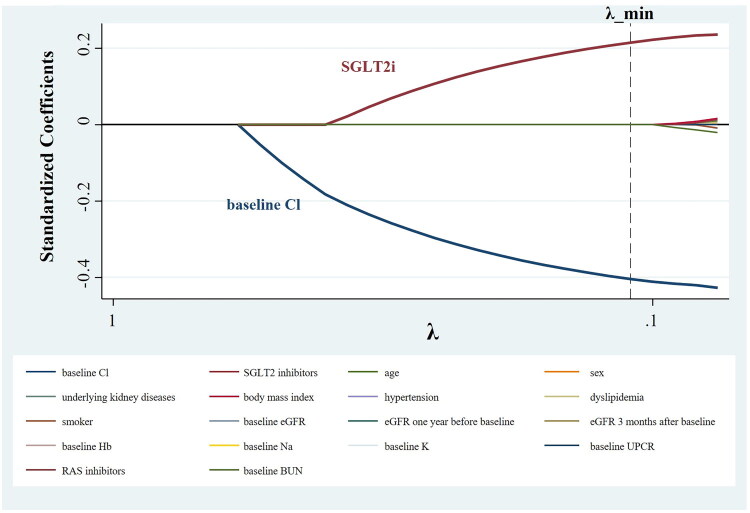
Coefficient paths of factors associated with mean changes in chloride levels in LASSO Cox regression. SGLT2i, sodium–glucose cotransporter 2 inhibitor. Among the evaluated variables, baseline chloride concentration and SGLT2i use demonstrated significant associations with the change in chloride concentration, with standardized coefficients of −0.404 and +0.215, respectively.

Multivariable analysis identified the baseline serum chloride concentration and the use of SGLT2is as independent factors associated with the change in serum chloride concentration, with coefficients of −1.84 (95% CI: −2.52 to −1.15) and 0.45 (95% CI: 0.08 to 0.82), respectively (Table 2). Next, we used a LASSO regression model to identify the factors associated with the change in serum chloride concentration from among 18 candidate variables, considering that the three eGFR variables exhibited multicollinearity. The factors identified were the baseline chloride concentration and SGLT2i use, with standardized coefficients of −0.404 and 0.215, respectively (Figure 5). These results suggest that the use of SGLT2is is independently associated with an increase in serum chloride levels.

**Table 2.** Results of the multivariable analysis to identify factors associated with the mean changes in chloride concentrations.

	Coefficient	95% CI	*p*
Age	0.45	−0.09 to 0.99	0.11
Sex (male)	−0.12	−0.47 to 0.22	0.48
**Etiology of CKD**			
Nephrosclerosis *vs.* glomerulonephritis	−0.01	−0.42 to 0.41	0.99
Others *vs.* nephrosclerosis	−0.08	−0.50 to 0.33	0.70
Body mass index	0.41	−0.13 to 0.96	0.14
Hypertension	−0.39	−0.76 to −0.02	0.04
Dyslipidemia	0.12	−0.22 to 0.46	0.50
Smoker	−0.12	−0.50 to 0.26	0.54
Baseline eGFR, mL/min/1.73 m^2^	−0.15	−0.91 to 0.60	0.69
Proteinuria, g/gCr	−0.04	−0.36 to 0.27	0.80
Sodium, mEq/L	0.17	−0.71 to 1.05	0.71
Potassium, mEq/L	−0.24	−0.86 to 0.37	0.44
Chloride, mEq/L	−1.84	−2.52 to −1.15	<0.001
Renin-angiotensin receptor blockers	0.26	−0.09 to 0.62	0.15
SGLT2 inhibitors	0.45	0.08 to 0.82	0.017

CI, confidence interval; CKD, chronic kidney disease; SGLT2, sodium–glucose cotransporter 2.

## Discussion

In the present study, we identified a significant increase in the chloride concentrations of patients with CKD but no diabetes who were being treated with SGLT2is *versus* those who were not. Regardless of the baseline chloride concentrations of the patients, SGLT2i administration resulted in a statistically significant increase in chloride concentration. Furthermore, subgroup analyses based on age, sex, and other variables yielded similar results, indicating the robustness of this finding. Conversely, there were no significant changes in the serum sodium or potassium concentrations after the initiation of SGLT2i treatment. While large outcome trials such as DAPA-CKD and EMPA-KIDNEY have focused on eGFR and cardiovascular outcomes, our findings suggest that chloride modulation may represent a complementary biochemical pathway underlying these benefits [5,6,25].

Chloride is the principal extracellular anion and plays key roles in maintaining osmotic pressure, acid–base balance, and fluid distribution [26]. It is absorbed throughout the intestine and predominantly excreted *via* the kidneys. Approximately 19,440 mmol of chloride is filtered daily by the glomeruli, of which more than 99% is reabsorbed, leaving only about 180 mmol excreted [27]. The proximal tubule reabsorbs around 60% of chloride, with little uptake in the early segments (S1, S2) and passive reabsorption in the later segment (S3). The loop of Henle reabsorbs 15–25% through the Na^+^/K^+^/2Cl^−^ cotransporter, and the distal convoluted tubule reabsorbs chloride *via* the Na^+^/Cl^−^ cotransporter, both regulated by with-no-lysine kinases. In the collecting duct, sodium reabsorption *via* ENaCs generates a negative potential that drives paracellular chloride flux, while β-intercalated cells mediate transcellular transport. The gastrointestinal tract also contributes to chloride homeostasis, partly through Na^+^/K^+^/2Cl^−^ cotransport [28].

Hypochloremia has been consistently shown to be associated with adverse clinical outcomes across a range of pathological conditions, including liver cirrhosis, sepsis, and heart failure [29–31]. Low chloride concentrations, defined as ≤103.9 mEq/L, were found to be significantly associated with all-cause mortality and cardiovascular events in patients with CKD before commencing dialysis [32]. In patients with CKD, the serum chloride concentration has substantial effects on the risks of incident atrial fibrillation, heart failure, stroke, and all-cause mortality [33]. Specifically, each 5-mEq/L decrease in serum chloride concentration is associated with a 22% increase in the risk of atrial fibrillation. In contrast, in patients with CKD, every 1-mEq/L increase in chloride concentration is associated with an overall decline in eGFR of 0.32 mL/min/1.73 m^2^ [34], implying that chloride concentration may be a useful predictor of CKD progression. As for heart failure, hypochloremia may contribute to a poor prognosis for patients with CKD through multiple pathophysiological mechanisms: activation of the renin–angiotensin system secondary to detection in the macula densa of the distal nephron [35], attenuation of the natriuretic response to loop diuretics, resulting in worsening congestion [36], and the development of metabolic alkalosis, which may predispose to arrhythmias and a reduction in cardiac output [37].

In the present study, patients with CKD but no diabetes or proteinuria who were taking a SGLT2i showed significant increases in serum chloride concentration over 2 years, in contrast to those who were not. The mechanism by which SGLT2is increase serum chloride concentration remains unclear, but compensatory mechanisms within the nephron may be involved in mitigating the dehydration and acidosis resulting from the inhibition of SGLT2 and Na^+^/K^+^ exchanger 3 (NHE3) in the proximal tubule by SGLT2is [38]. SGLT2is have been shown to upregulate uromodulin expression, which in turn activates Na^+^/K^+^/2Cl^−^ cotransporters in the thick ascending limb of the loop of Henle [39]. The high aldosterone and luminal glucose concentrations induced by SGLT2 inhibition may contribute to the activation of Na^+^/Cl^−^ cotransporters in the distal convoluted tubule [40,41]. The upregulation of these channels to enhance sodium reabsorption necessitates concomitant chloride reabsorption. In addition, in compensation for the reduction in bicarbonate reabsorption induced by NHE3 inhibition by SGLT2is [42], the high concentrations of α-ketoglutarate in the proximal tubule facilitate chloride reabsorption through the activation of chloride/bicarbonate exchange pathways. In parallel, α-ketoglutarate enhances ammoniagenesis, thereby promoting renal acid excretion [43].

There are several points that should be addressed regarding this study. First, the reason for the slight decline in serum chloride concentration (approximately −1 to −0.5 mEq/L) observed in the SGLT2 non-user group remains unclear. In this study, we confirmed that no diuretics were used at baseline or during the follow-up period. Possible explanations include dietary salt restriction following multidisciplinary intervention or a subclinical increase in extracellular fluid volume that is not clinically detectable [44]. Second, in the subgroup analysis, changes in chloride concentration were evaluated using 104 mEq/L as the cutoff value. Among patients with chloride levels ≤104 mEq/L, SGLT2 is significantly increased chloride concentrations. Although a chloride level ≤104 mEq/L is not generally defined as hypochloremia, these findings suggest that SGLT2 inhibitors corrected slightly lower chloride levels within the normal range. Third, it could also be argued that a rise in chloride concentration without a concomitant change in sodium might promote acidosis. Nevertheless, in this study, baseline chloride concentrations were ≤104 mEq/L, with an observed increase of only about 1.5 mEq/L; when the baseline was below 104 mEq/L, chloride concentrations were essentially maintained. Thus, these results reflect only a minor alteration in chloride concentration, confined well within the physiological range of normal pH.

Fourth, several limitations of the present study should be noted. First, only patients with CKD but no diabetes or proteinuria were studied. Most patients with chronic glomerulonephritis in this study had chronic lesions that persisted after treatment with steroids and immunosuppressive agents, leading to less severe proteinuria than that observed in previous studies. Therefore, it remains to be determined whether SGLT2is increase the serum chloride concentrations of patients with diabetic kidney disease or CKD accompanied by proteinuria. Second, because diuretics may affect the serum chloride concentration, patients who were taking these drugs were excluded. Therefore, the findings may not be generalizable to patients who are undergoing diuretic therapy. Third, because it was a single-center study with a relatively small sample size, the possibility of selection bias cannot be excluded. To minimize this, 1:1 PS matching was employed; however, one-to-many propensity score matching could not be performed because of the limited number of eligible controls and post-matching subgroup analyses may have limited statistical power due to the reduced sample size. Therefore, we presented both pre- and post-matching results, which consistently demonstrated the effect of SGLT2i in maintaining chloride concentrations. Fourth, there may have been unmeasured confounding variables that could have affected the serum chloride concentration. Fifth, the addition of other concomitant medications, including RAS inhibitors, during the study period could not be tracked. Sixth, the occurrence of cardiovascular events was not assessed in this study; therefore, the association between chloride concentration and cardiovascular outcomes could not be established. One of the major strengths of the study is that it focused on a group of patients in whom there have been few studies of the use of SGLT2is. In these patients, tubular function can be assessed under relatively physiological conditions, and therefore, there were minimal confounding effects of ion and solute reabsorption and secretion. Furthermore, the validity and robustness of the results were supported by analyses following PS matching and several subgroup analyses.

In conclusion, SGLT2is have the beneficial effect of preserving serum chloride concentrations in patients with CKD but no diabetes or proteinuria. We propose that this effect on chloride concentration may, at least in part, contribute to the cardioprotective effects of these agents.

### Clinical and research implications

Our findings highlight serum chloride as a potential physiological biomarker of SGLT2 inhibitor activity in non-diabetic CKD. Future prospective studies integrating urinary chloride excretion, tubular transporter profiling, and acid-base balance may clarify whether modulation of chloride homeostasis contributes to improved cardiovascular outcomes. If validated, serum chloride could serve as a simple and inexpensive surrogate marker for SGLT2i response in routine CKD management.

## Supplementary Material

Supplementary Figure.docx

## Data Availability

The datasets that support the findings of the current study are not publicly available due to privacy reasons but are available from the corresponding author on reasonable request.
