# Supplementary material for: SGLT2 inhibitors preserve serum chloride in non-diabetic CKD: a propensity-matched and LASSO regression analysis
Source: Ren Fail. 2026 Feb 9;48(1):2624169. doi: 10.1080/0886022X.2026.2624169 (PMC12893160; doi:10.1080/0886022X.2026.2624169)
Supplement: Supplementary Figure.docx [file IRNF_A_2624169_SM4790.docx]

**Supplementary Figure 1**


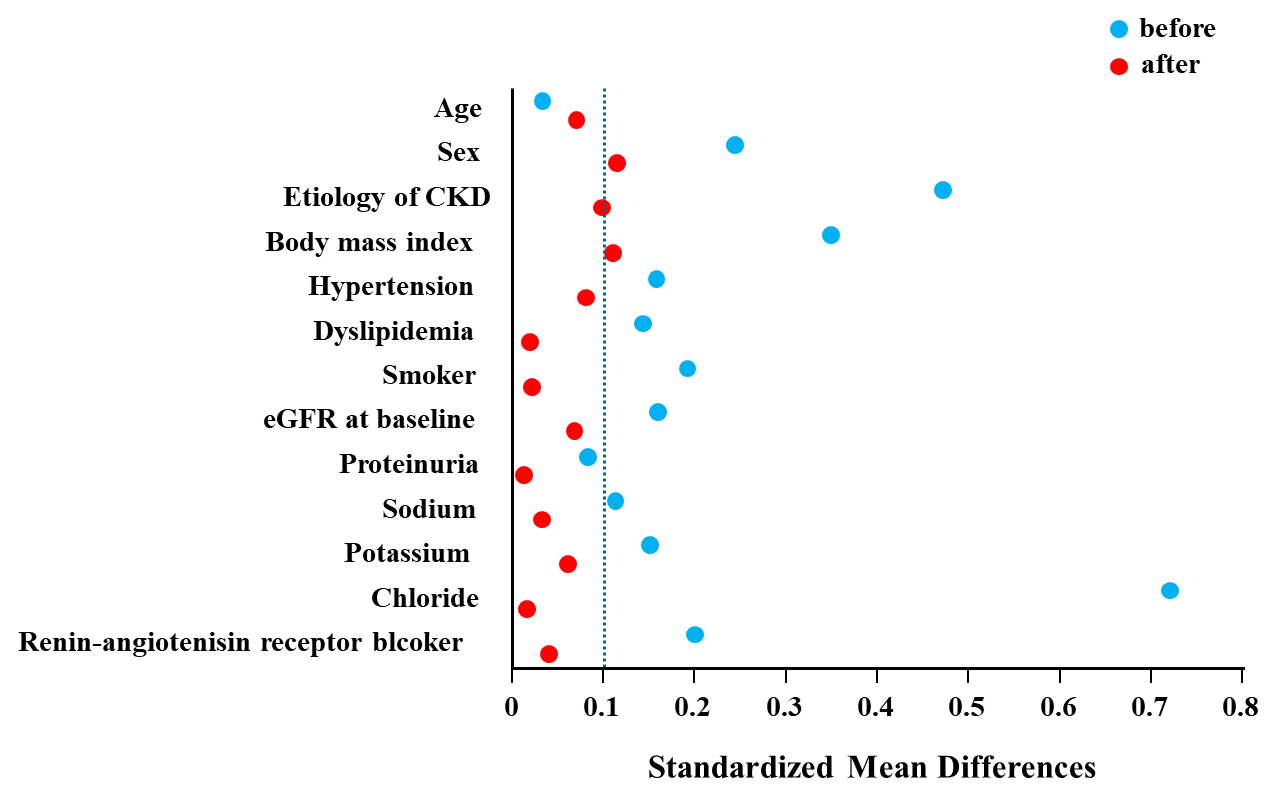


**Supplementary Figure 1. Love plot depicting standardized differences before and after propensity score matching.**

We confirmed that each covariate was well-balanced before and after propensity score maching.

**Supplementary Figure 2**


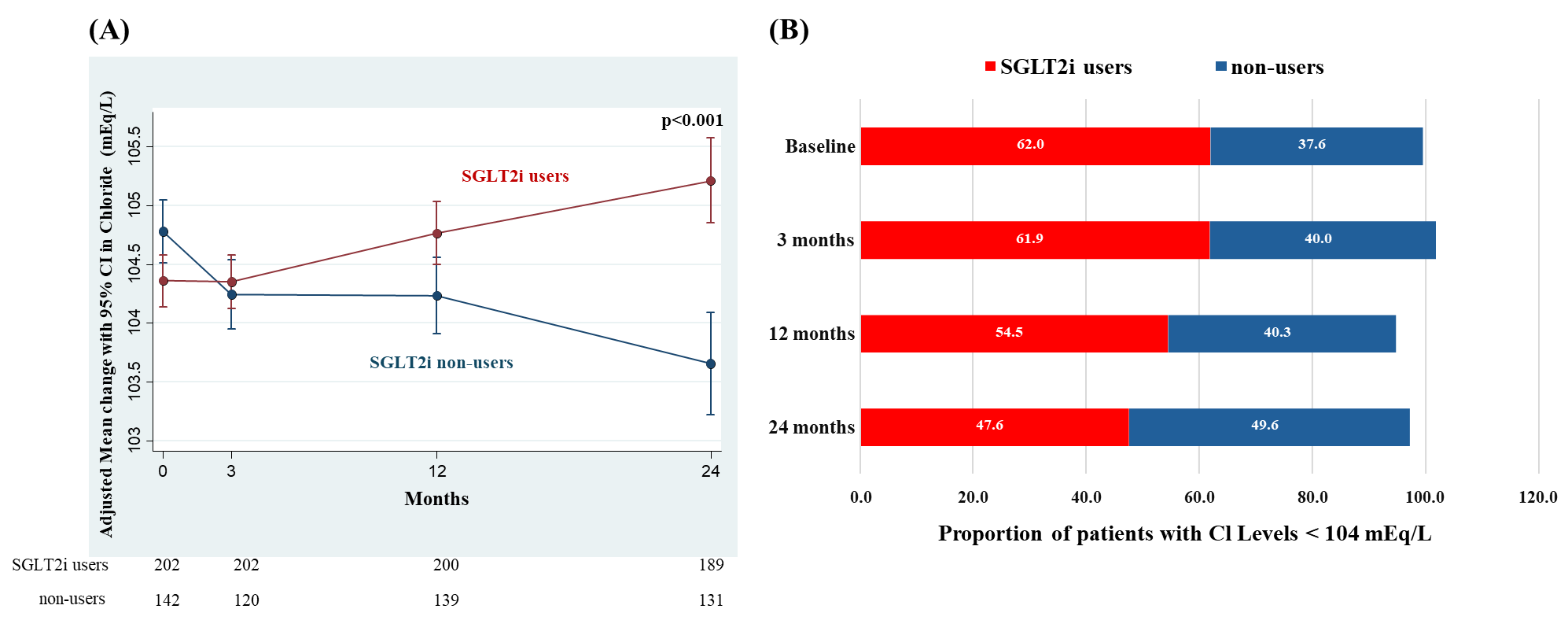


**Supplementary Figure 2. Adjusted change in chloride in SGLT2i users and non-users with common normal values.**

The SGLT2 users significantly preserved serum chloride concentrations compared with the non-users (A). Furthermore, as the study progressed over two years, treatment with SGLT2 inhibitors was associated with a gradual decline in the proportion of patients exhibiting chloride levels below 104 mEq/L (B).
